# Histomorphometric evaluation of 3D printed graphene oxide-enriched poly(ε-caprolactone) scaffolds for bone regeneration

**DOI:** 10.1016/j.heliyon.2023.e15844

**Published:** 2023-05-05

**Authors:** Maha H. Alazab, Salma A. Abouelgeit, Moustafa N. Aboushelib

**Affiliations:** a Menoufia University Hospitals, Menoufia University, Egypt; b Dental Biomaterials Department, Alexandria University, Egypt

**Keywords:** Bone regeneration, Scaffolds, Graphene oxide, Poly(ε-caprolacone)polymer, 3D printing, PRP

## Abstract

**Objective:**

Restoring large boney defects using bone grafts alone is an unpredictable procedure. Biodegradable polymeric scaffolds suffer rapid biodegradation and lack sufficient osteo-conductivity. The aim of this study was to histomorphometrically evaluate three-dimensional printed graphene oxide-enriched poly(ε-caprolactone) (PCL) scaffolds for bone regeneration in a rabbit defect model using two different concentrations of graphene oxide. Basic characteristic properties and mount of new bone regeneration formation were evaluated.

**Methods:**

two concentrations of graphene oxide (1 and 3 wt%) were added to PCL scaffolds using hot blind technique while pure PCL scaffolds served as a control. Laboratory characterization included scanning electron microscopy (SEM), x-ray diffraction analysis (XRD), contact angle, internal porosity, in addition to density measurements. All scaffolds were subjected to biodegradation evaluation and cell cytotoxicity test. In vivo bone regeneration was evaluated in the tibia defect of a rabbit model by measuring the amount of new bone formation (n = 15, ά = 0.05).

**Results:**

SEM images showed slight reduction in pore size and increase in filament width of scaffolds with increasing GO contents. However, the printed scaffolds matched well with the dimensions of the original design. XRD patterns revealed characteristic peaks identifying microstructure of scaffolds. Addition of GO increased crystallinity of the scaffolds. The contact angle and porosity readings indicated reduction in measurements with increased content of GO indicating improved wetting properties while the density followed an opposing pattern. Higher biodegradability values were associated with higher GO content resulting in acceleration of observed biodegradation. The results of cytotoxicity test showed reduction in cell viability with higher GO content. Bone regeneration was significantly enhanced for 1 wt% GO scaffolds compared to other groups as was evident by higher bone density observed in x-ray images and higher amount of new bone formation observed at different time intervals.

**Significance:**

Graphene oxide improved the physical and biological properties of PCL scaffolds and significantly enhanced new bone regeneration.

## 1 Introduction

While small bone defects could heal spontaneously, larger defects caused by accidents, injuries, infections, bone cancer surgeries, and chronic health conditions, represent relevant clinical problems that require surgical intervention to bridge the skeletal defect [1]. Current clinical therapies, based on the use of biological grafts, have their drawbacks such as restricted quantity and availability of donor bone, pain and morbidity in grafting site, deep infection and risk of hematoma, rejection, and disease transmission.

Scaffolds are three-dimensional (3D) porous physical substrates used for supporting the graft material and designed to enhance cell attachment, proliferation, and differentiation [[2], [3], [4]]. They must be biocompatible with the host tissues, biodegradable at an appropriate rate which matches the rate of new tissue formation, have adequate porosity and density, surface characteristics, and mechanical properties [5]. Another important requirement, is the capacity to stimulate target cells. Due to the piezoelectric and reverse piezoelectric nature of bone, electrical signals are considered as a critical physiological stimuli that has strong effect on cell behavior controlling their migration, adhesion, growth, and differentiation [6]. A wide range of polymers (e.g. collagen, chitosan, polylactic acid, polyglycolic acid, poly(ε-caprolactone), and polylactide-co-glycolide), and various ceramic materials (e.g., hydroxyapatite, bioactive glass, and β-tricalcium phosphate), and composites of the upper mentioned materials have been used to produce bone scaffolds [[7], [8], [9]].

In order to produce electro-active scaffolds, different routes were explored, including mixing non-conductive polymers with conductive ones, or mixing non-conductive polymers with inorganic conductive fillers [10,11]. Graphene oxide (GO), a single monomolecular layer of graphite, has recently attracted substantial attention as a bioactive material used to promote cell proliferation and differentiation [12]. It is a has numerous functionalities due to presence of carboxyl, hydroxyl, epoxide and carbonyl groups and is considered a candidate material for fabrication of electro-active scaffolds [13]. The effect of addition of GO to different polymeric matrices was thoroughly investigated.

Poly(ε-caprolactone) polymer (PCL), on the other hand, is a biocompatible, biodegradable, and strong synthetic polymer which has a wide number of advantages compared to the other synthetic and natural polymers. However, it has low cellular affinity because of its hydrophobic nature, slow degradation rate extending from 24 up to 36 months in human body, and low mechanical properties [14]. Rapid biodegradation and lack of sufficient osteo-conductivity is a problem facing several polymeric based scaffolds. Early chemical disintegration of the polymeric matrix increases the risk of fibrous tissue formation while poor osteo-conductivity increases the risk of poor bone formation and integration.

The addition of GO to PCL scaffolds might improve their structural properties and enhance their osteogenic potential. No data are currently available about suitability of 3D printing technology for manufacturing porous and structured PCL-GO biodegradable scaffolds. The aim of this study was to histomorphometrically evaluate the effect of addition of two concentrations of GO (1 and 3 wt%) to porous 3D printed PCL scaffolds. Different characterization techniques were used to assess expected performance of the scaffolds. The proposed hypothesis was that addition of GO would enhance bone formation in an animal model.

## 2 Methods

In this study, a polymeric based PCL porous scaffolds were manufactured using 3D printing additive manufacturing technique. Scaffold internal dimensions were controlled using G code language for controlling the printing head movements. Two concentrations of GO were added to the scaffolds to improve their mechanical and biological properties. After laboratory characterization, scaffolds were implanted in femur of a rabbit model, and new bone formation was histologically evaluated. Unmodified PCL scaffolds served as a control.

### 2.1 Fabrication of 3D printed scaffolds

Poly(ε-caprolactone) (Mn = 80,000, Sigma‐Aldrich Co. Ltd., St. Louis, MO, USA) and graphene oxide (Sigma‐Aldrich Co.) mixtures were prepared by hot melt-blending process using two different graphene oxide concentrations (1 and 3 wt%). Briefly, pure PCL pellets were heated above 70 °C to ensure that the polymer was homogenously melted before graphene oxide powder was added at the required concentrations. The materials were then mixed over night to get a homogenous mixture and left to cool down to room temperature then finally cut into small pieces [15]. Pure PCL was used as a control.

**Fig. 1:**
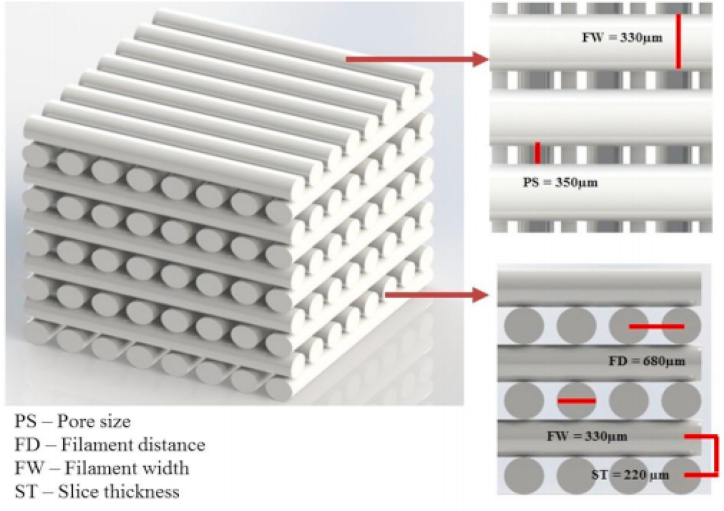
Design parameters of 3D printed scaffolds.

The designed scaffolds had two important design parameters, the first is related to its external geometry, which was controlled using a computer assisted design software (Materialise mimics 20, Materialse) to manufacture cylindrical implant shaped specimens. Secondly, which is the design related to the internal porosity of the printed scaffolds. This was achieved by controlling printing head nozzle movement through G code machine language. The blended materials were introduced into a single screw extruder (Noztek Pro HT, England) to fabricate 1.75 mm diameter continuous filaments suitable for 3D printing. The G-code for nozzle movement was written to print a 3D network in a 0°–90° pattern providing the following design parameters: filament width (FW) of 330 μm, pore size (PS) of 350 μm, while maintaining a constant filament distance (FD) of 680 μm and slice thickness (ST) of 220 μm, Fig. 1 [15]. The final dimensions of the printed scaffolds was 30 × 30 × 4 mm which were printed using the following parameters using (Prusa i3 Hephestos 3D printer); nozzle temperature of 130 °C, printing speed of 500 mm/min, and fan speed cooling speed of 100 hz. Cylindrical implants were similarly produced (3 mm in diameter x 6 mm in length). Random samples were collected from each group (n = 12) using random computer-generated numbers.

### 2.2 Characterization of scaffolds

Scanning electron microscopy (SEM) was used to examine the internal structure of the printed scaffolds, measure pore size and filament size. Scaffolds were gold sputter coated and examined at different magnifications. Cross-section images were also used to study internal architecture of the specimens. Obtained values were compared with the initial design dimensions [16]. X-ray diffraction (XRD) was used to obtain information about microstructure and crystallinity of scaffolds using X-ray diffractometer (Philips PW3710) equipped with a Cu−Kα radiation, and operated at 50 kV and 40 mA [17]. According to the radiation used, CuKα1, wavelength 1.54, the obtained peaks were identified using ICDD grant-in-aid publications of Institut fur kristallgraphie dr RWTH, Aachen, Germany.

Static contact angle measurements were performed by a precise goniometer (DSA100, Data physics Instruments, Germany) using the sessile drop method. Deionized water droplets of ∼4 μL were deposited via a motorized syringe at a velocity of 1 μL/s and the drop shape was recorded with a highspeed framing camera, 10 s after droplet addition. Five measurements per specimen were performed to determine the static contact angle. Contact angle was used as a measure of surface liquid wetness.

Porosity and density measurements were recorded by liquid displacement test using ethanol as the liquid. With a graduated cylinder that contains a known ethanol volume (V1), the scaffold of a known weight (W) was immersed for 5 min. The total ethanol volume in the cylinder containing the impregnated scaffold was recorded as (V2). The impregnated scaffold was then removed and the residual ethanol volume in cylinder was recorded as (V3). For each scaffold, five measurements were averaged. The porosity (Є) and the density (d) of the scaffolds were calculated using the following formulas [18]:

Є = (V1–V3)/(V2–V3). d = W/(V2–V3)

### 2.3 In vitro biodegradability

The weight loss was measured in correspondence to time to evaluate the rate of biodegradation. After measuring the dry weight, the scaffolds were immersed in 45 mL of phosphate buffered saline (PBS, Biowest Co, USA) at 37 °C and rotated at a speed of 50 rpm in a thermoshaker device (LS‐100, Thermo Scientific) for 14, 21 and 28 days. At the end of each week, the scaffolds were dried and weighed again. The biodegradation percentage of the scaffolds was calculated by the following equation in which W0 is the dry weight and W is the weight of specimen after specific soaking time [19].

Biodegradation ratio (%) = [(W–W0)/W0] × 100

### 2.4 Cytotoxicity evaluation

The MTT cell viability assay was used to evaluate cell survival by reduction of a tetrazolium salt solution MTT (3-(4,5-dimethylthiazolyl-2)-2,5-diphenyltetrazolium bromide) to insoluble purple formazan crystals by all metabolically active cells. The viability and proliferation rates of human peripheral blood mononuclear cells (PBMCs) in direct contact with the prepared scaffolds was determined using the MTT spectrophotometric assay after 1, 2 and 3 days of cell culture. The culture media was discarded and the cells were incubated for 4 h with 1 mg/mL MTT solution, in standard conditions. The resulted formazan crystals were then solubilized in isopropanol and the solution concentration was determined by spectrophotometry at 550 nm. The relative cell viability (%) compared to control wells was calculated using the following equation, where (A) is the absorbance and the control were wells containing cells without the scaffolds [17,20]:

Cell viability % = (A) test/(A) control × 100

### 2.5 Histomorphometric analysis

Cylindrical scaffolds were sterilized with UV radiation before implantation [21]. New Zealand male white rabbits, 6 months old, were used. The surgical procedures were performed according to the guidelines set by the Ethics Research Committee of the Faculty of Dentistry, Alexandria University, Egypt (IRB No.: 00010556-IORG 0008839). The surgical procedures were conducted under proper general anesthesia using 0.35 ml kg^−1^ ketamine chlorhydrate and 0.25 ml kg^−1^ xylazine chlorhydrate (Francotar, Virbac, Brazil). The fur was trimmed, followed by skin disinfection with surgical iodine solution (Nile-pharma, Egypt). A 2 cm rectilinear incision was made under the tibia tuberosity at the upper end of tibia. The adjacent soft tissue was reflected to expose bone, the periosteum was incised, and a tibial perforation was performed using a 2 mm pilot drill, cooled with a physiological saline solution at 700 rpm. A sequential 2.5 and 3 mm drills were used to reach final implant dimension. The scaffolds were then placed using press-fit technique. Control defects were left empty. The periosteum was repositioned followed by wound suturing, Fig. 3 [22]. Rabbits were given 0.2 ml of penicillin subcutaneously and ketoprofen (3 mg/kg body weight) intramuscularly for 5 days post-surgery (Eipico pharma, Egypt). After four and eight weeks, the rabbits were euthanatized by CO^2^-inhalation and prepared for radiographical, and histomorphometric examinations to evaluate bone regeneration potential in acrylic embedded sections (Loba chemie Ltd., India). In histomorphometric examination, the amount of stained new bone percentage obtained from light microscope images was measured using the image analysis system (image J, the mathworks, inc., USA) and divided by the surface area of field (7047200 pixel) X 100) [23].

All date were digitally analyzed (SPSS 20, SPSS inc) using one way and two-way analysis of variance and Bonferroni post hoc tests (ά = 0.05, n = 15). Data were checked for homogeneity, equal distribution, and presence of outliers. According to small effect size difference, the power of the selected test was 0.9. Sample size calculations were performed using z-score formula.

## 3 Results

**Fig. 2:**
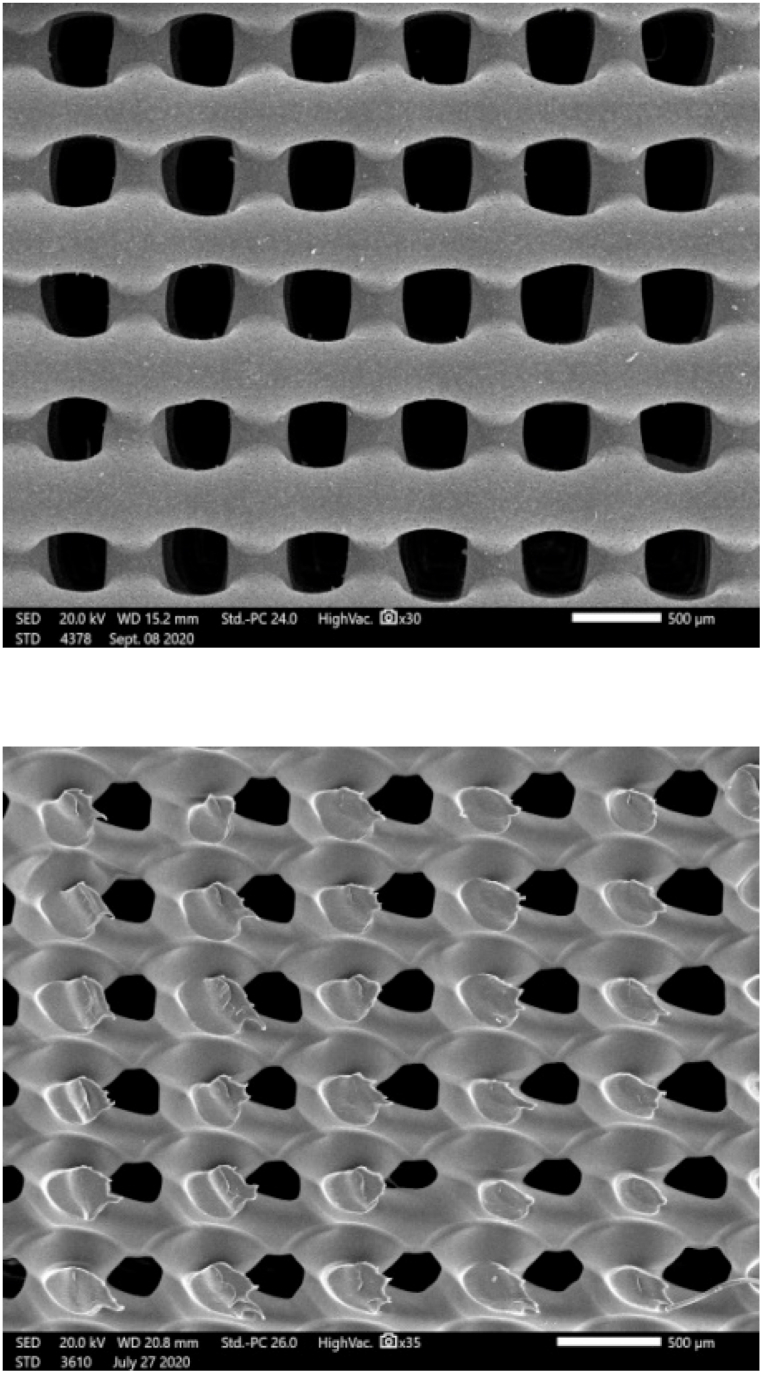
Top surface and cross-section SEM images of 3D printed scaffolds.

SEM images revealed that the printed scaffolds matched closely (F = 138, *P* < 0.01) the intended design with a well-defined internal geometry and uniform pore distribution. Top view images revealed even spacing of scaffold filaments while cross-section micrographs showed good adhesion between adjacent layers, Fig. 2. The values of pore size and filament width are summarized in Table 1. Cross-section images revealed proper integrity and coherence of the printed layers without presence of delamination or separations between the filaments.

**Fig. 3:**
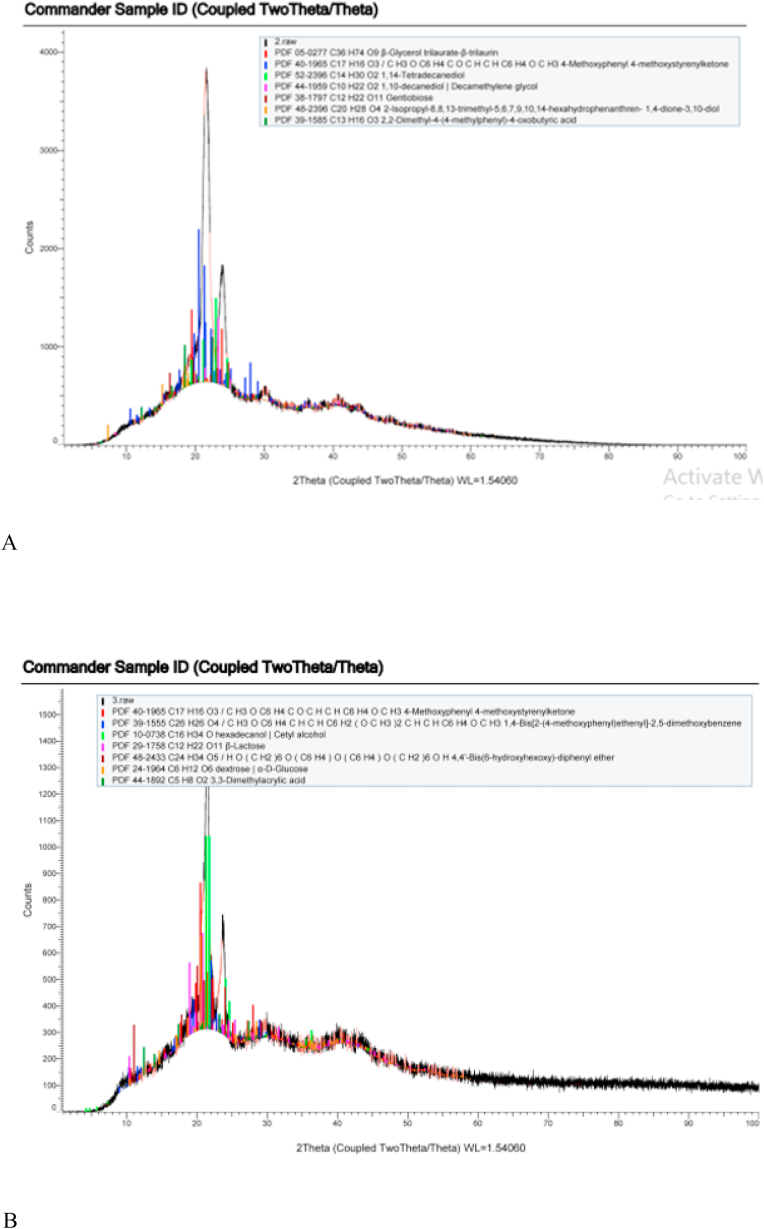
XRD patterns of (A) pure PCL scaffolds, (B) 3 wt% GO-PCL scaffolds.

**Table 1:** Characterization measurements of tested groups.

Scaffolds	Filament width (μm)	Pore size (μm)	Contact angle°	Porosity (%)	Density (g/cm3)	Cell viability %
PCL	326 ± 20.8	347 ± 22.55	94 ± 10.44	56 ± 3.7	0.24 ± 0.016	83.27 ± 5.55
1 wt% GO-PCL	332 ± 21.66	339 ± 22.72	87 ± 9.67	52 ± 3.5	0.26 ± 0.017	86.34 ± 5.76
3 wt% GO-PCL	335 ± 21.36	335 ± 20.01	83 ± 9.22	50 ± 3.3	0.27 ± 0.018	79.36 ± 5.29

XRD patterns revealed two PCL characteristic peaks at 2θ = 21.9° and 2θ = 23.5° due to its carbon and hydrogen contents which correspond to the (110) and (200) crystallographic planes respectively and a third peak at 2θ = 40.1° due to its oxygen content. These peaks were observed in the composite scaffolds as well but with higher peak intensities as GO resulted in slight increase in crystallinity of the composite scaffolds, Fig. 3A and B. Peaks were compared to standard data cards to identify the materials and identify related crystallographic structures using qualitative phase analysis method.

**Fig. 4:**
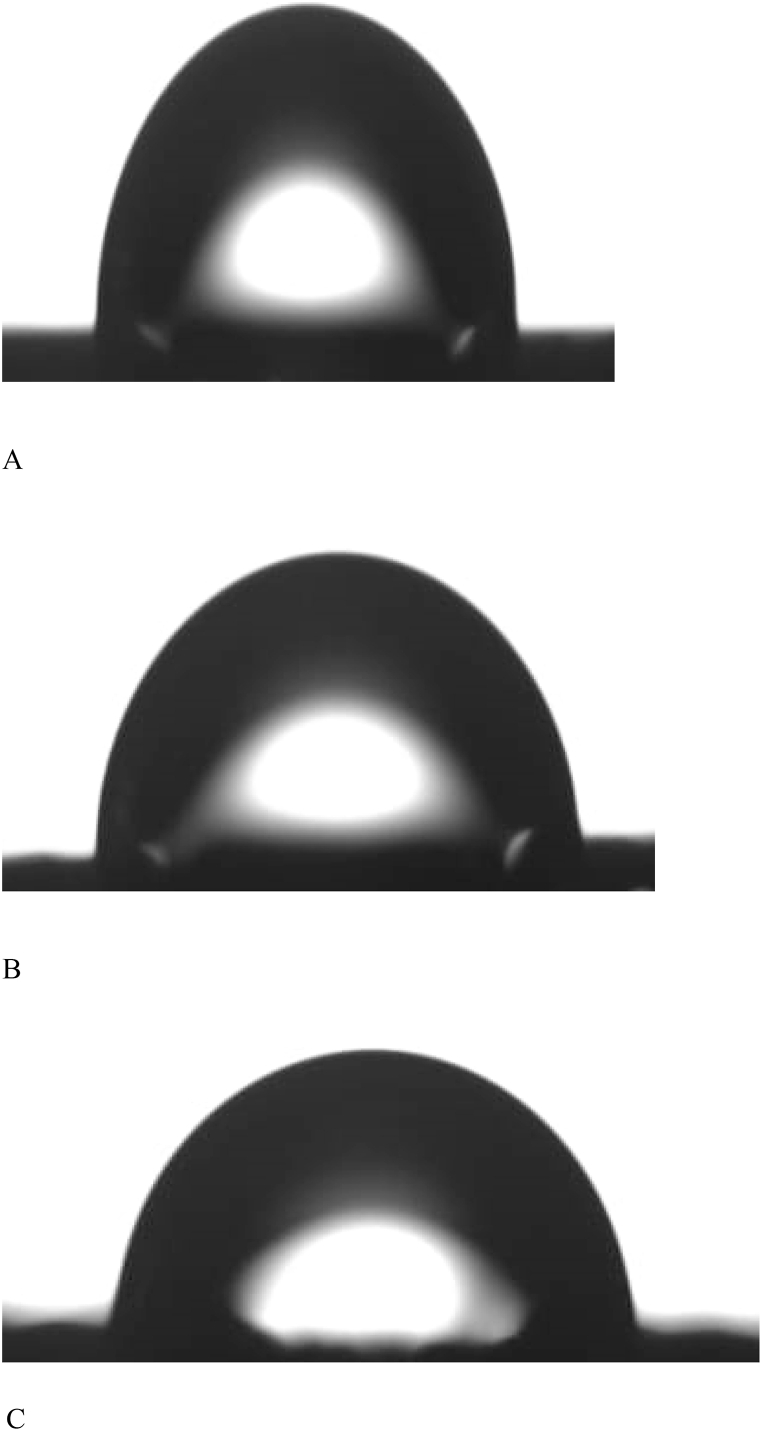
Contact angle of pure PCL (A), 1% GO (B), and 3% GO.

Static contact angle measurements showed a significant decrease in measured values (F = 98, *P* < 0.014) by increasing GO concentration with a marked shift from hydrophobic PCL scaffolds to hydrophilic composite ones, Table 1 and Fig. 4A–C. Decrease in contact angle is a direct reflection of improved wetting properties of the scaffolds.

Porosity of scaffolds did not change significantly (F = 11.2, *P* < 0.174) with increasing GO content, neither did the measured densities (F = 11.7, P < 0.69) which indicate proper incorporation of GO powder within the filament structure of the scaffolds. Addition of GO, also resulted in significant increase (F = 123, *P* < 0.001) in degradation rate of PCL scaffolds, Table 2. No marked changes in cell cytotoxicity were observed between all tested groups, Table 1. The count of living cells was statistically comparable between all tested groups.

**Table 2:** Weight loss and biodegradation measurements.

	W0	W after 14 days	Biodegradation %	W after 21 days	Biodegradation %	W after 28 days	Biodegradation %
PCL	0.251 ± 0.02	0.238 ± 0.02	5.2 ± 0.47	0.226 ± 0.02	9.96 ± 0.91	0.214 ± 0.02	14.7 ± 1.34
1 wt% GO-PCL	0.302 ± 0.03	0.274 ± 0.02	9.3 ± 0.85	0.247 ± 0.02	18.2 ± 1.65	0.221 ± 0.02	26.8 ± 2.44
3 wt% GO-PCL	0.328 ± 0.03	0.292 ± 0.03	11.0 ± 1.00	0.258 ± 0.02	21.3 ± 1.94	0.225 ± 0.02	31.4 ± 2.85

**Fig. 5:**
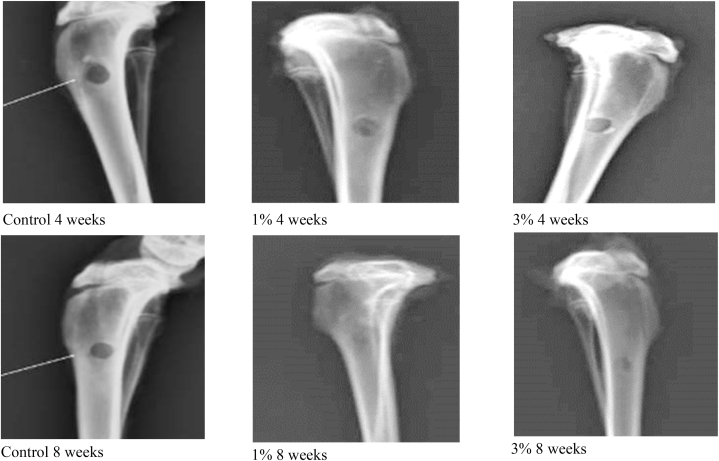
Digital x-ray photographs of rabbit tibias bone defects after 4 weeks (top raw) and 8 weeks (bottom row) of healing (from left to right, control, 1% and 3% GO-PCL scaffolds). Highest amount of bone formation was associated for 1% GO-PCL after 8 weeks of healing.

Digital radiographic images of the dissected rabbit's tibias after 4 and 8 weeks of implantation time showed that the control group was associated with a radiolucent defect with minimal new bone regeneration as was evident by the amount of radiolucency surrounding defect size. Highest wound healing was associated with 1 wt% followed by 3 wt% GO-PCL scaffolds which demonstrated higher bone density surrounding the bone defects, Fig. 5. Amount of radiolucency decreased over different test intervals indicating increase in bone density with time.

**Fig. 6:**
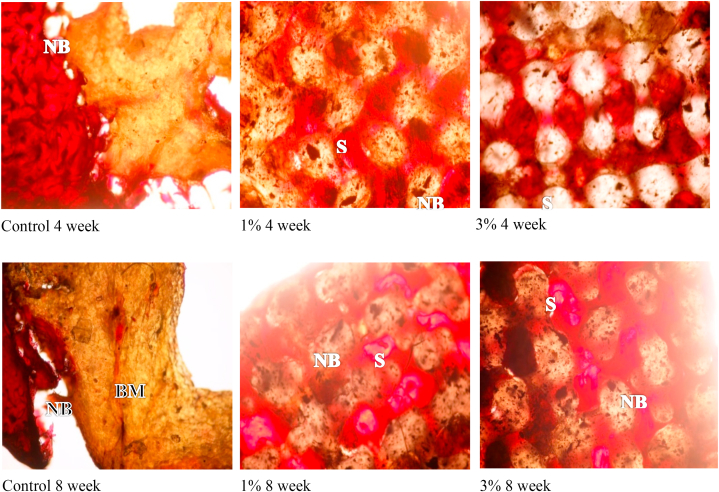
Light microscope histological images of scaffolds after 4 weeks, top raw, and 8 weeks, bottom raw, of healing time. (from left to right, control, 1% and 3% GO-PCL scaffolds). NB: New Bone. OB: Old Bone. BM: Bone Marrow. S: Scaffolds.

Histomorphometric analysis, revealed that 1 wt% GO-PCL scaffolds showed superior bone healing (F = 38.1, *P* < 0.037) compared to 3 wt% GO-PCL scaffolds while the controls were associated with the least amount of bone formation at all measured time intervals. Amount of new bone formation was in correlation with the rate of scaffold biodegradation as no gaps were detected in any of the examined sections. All specimens showed de novo bone formation at the surfaces of the scaffolds. No signs of pathology, inflammatory reactions, foreign body reactions or fibrous encapsulation were observed. Four weeks after implantation, moderate regenerated bone tissue was detected inside the pores of GO-PCL scaffolds. Significant increase in the amount of new bone formation was observed after 8 weeks for all PCL scaffolds enriched with GO, Fig. 6 and Table 3.

**Table 3:** Percentage of new bone formation at different time intervals.

	Implantation time	Average area of new bone	New bone %
Control	4 weeks	1183930	16.8 ± 1.53
	8 weeks	1430582	20.3 ± 2.26
1 wt% GO-PCL	4 weeks	3086674	43.8 ± 4.38
	8 weeks	3692733	52.4 ± 4.03
3 wt% GO-PCL	4 weeks	2952777	41.9 ± 3.49
	8 weeks	3565883	50.6 ± 5.62

## 4 Discussion

In this study, PCL based scaffolds were enriched with two concentrations of GO using hot blending method which requires careful heating and handling to obtain a homogenous mixture. Solvent blending could also be used with easily dissolved polymers. The selected characterization techniques offer a broad insight about the general properties of the scaffolds; however, further investigations are required to reveal expected interaction with the surrounding environment. Histomorphometric data could be further investigated using specific staining techniques. Additionally, different concentrations of GO could be tested with a variety of composite polymers and piezoelectric behavior could be studies to investigate its effect on bone regeneration.

Scaffolds could be described as ‘‘the beating heart” of the tissue engineering field. The growth of cells in an artificial environment could not be perfectly controlled without using the appropriate scaffold especially in location subjected to load bearing which might cause collapse of the graft material. The aim of this study was to investigate the influence of adding two small concentrations of graphene oxide to 3D printed PCL scaffolds on bone regeneration potential in a rabbit defect model. SEM micrographs showed that the 3D printed scaffolds had a well-defined internal geometry and uniform pore distribution and dimension which is favorable for bone cell ingrowth. Minimum pore size for bone growth was estimated to be in range of 100–135 μm. The printed dimensions matched perfectly with the intended design parameters thus more sophisticated designs are expected to be printed with high quality. The selected printing parameters resulted in coherent scaffolds without presence of separation or delamination between the printed filaments.

XRD calculated peaks gave insight about the crystallinity of the printed scaffolds which increased with addition of GO, Fig. 3A and B. The results are in agreement with Castilla-Cortázar et al. who used XRD to characterize the surface chemical composition of PCL/GO hybrids [24]. Water contact angle measurements showed that pure PCL scaffolds were associated with the highest values, which is typical for hydrophobic surfaces. The reason for the hydrophobicity of PCL could be attributed to the CH2 groups in the main chain of PCL [19]. This hydrophobicity is not favorable for the attachment and proliferation of cells [25]. Addition of GO resulted in marked shift to a more hydrophilic surface improving wettability which is a direct attribute to the hydrophilic O, OH, and COOH groups of the GO nanosheets [16]. Reduction of contact angle is a direct measure for improved wetting of the scaffolds, Fig. 4A–C. Neither density or porosity measurements were affected by addition of GO which indicate that addition of GO did not interfere with 3D printing nor did affect melting, flow, and adhesion of the printed filaments [26,27].

The degradation rate of the scaffolds should match the rate of formation of new bone to allow an efficient load transfer to the healing defect. A degradation rate extended over 2–3 months is considered optimal for bone formation [28]. PCL has an extended degradation rate due to its hydrophobicity and crystallinity [29]. Addition of GO significantly increased biodegradation of the prepared scaffolds by almost 20%/month which provides sufficient time for bone deposition and remineralization. Incorporation of GO, added hydrophilic groups which in turn increased the capacity for absorbing surrounding fluids [19,30].

Regarding cytotoxicity, after the third day of cell seeding, 3 wt% GO-PCL scaffolds showed lower average cell viability% than the other groups. These results are in agreement with Hou et al. who added different concentrations of GO to PCL scaffolds for bone repair applications and reported potential cytotoxicity effects particularly for high levels of GO (3 wt%) [16]. According to ISO 10993–5(E), these values of cell viability still remained within the range of accepted level (cell viability>70%) [31].

X ray and histomorphometric data of GO-PCL scaffolds supported their biocompatible and osteogenic nature. The results of radiographic examination showed minimal new bone regeneration for empty defects, while highest amount of new bone formation was associated with 1 wt% GO-PCL scaffolds showing better results than 3 wt% GO-PCL scaffolds after 4 and 8 weeks. Histomorphometric assessment quantified the amount of newly formed bone area showing that bridging of a large bone defect without a scaffold was problematic. Insertion of GO-PCL scaffolds seemed to successfully solve this matter. The average amount of newly formed bone for 1 wt% GO-PCL scaffolds was the highest at 8 weeks observation time. Slight reduction in average newly formed bone area for 3% GO-PCL scaffold might be correlated to the results of cytotoxicity test that showed a lower cell viability% with increasing GO concentration.

Several studies have evaluated modifying polymeric scaffolds using GO and other calcium based mineral fillers. Different advanced manufacturing techniques were also investigated but controlling the movement of the printing nozzle enabled precise reproduction of the required internal porosity in 3D space which is considered a direct advantage in the field of bone regeneration [[32], [33], [34], [35], [36]].

## 5 Conclusions

Addition of GO to PCL scaffolds using hot blind technique allowed accurate manufacturing of porous integrated GO enriched PCL scaffolds using 3D printing technology. The manufactured scaffolds matched the intended design regarding pore size, filament width, and integration of successive layers. Addition of GO did not interfere with the 3D printing technique and resulted in improving biodegradability and wetting properties of PCL scaffolds. These changes were directly associated with rapid healing of wound defects and higher amount of new bone formation as were observed from x-ray images of histological sections obtained from the surgically created tibial defects.

## Author contribution

Maha H. Alazab: performed the experiments.

Salma A. Abouelgeit: conceived and designed the experiments.

Moustafa N. Aboushelib: analyzed and interpreted the data, contributed to reagents, materials, analysis tools or data, and wrote the paper.

## Data availability statement

Data included in article/supp. material/referenced in article.

## Declaration of competing interest

The authors declare that they have no known competing financial interests or personal relationships that could have appeared to influence the work reported in this paper.
